# A cerebellar learning model that reproduces the behavior of vestibulo-ocular reflex adaptation in wild-type and knock-out mice

**DOI:** 10.1186/1471-2202-14-S1-O4

**Published:** 2013-07-08

**Authors:** C Clopath, A Badura, CI De Zeeuw, N Brunel

**Affiliations:** 1 UMR 8118, CNRS and Université Paris Descartes, Paris, France; 2 Center for Theoretical Neuroscience, Columbia University, New York, USA; 3 Netherlands Institute for Neuroscience, Royal Dutch Academy of Arts and Sciences, Amsterdam, The Netherlands; 4 Department of Neuroscience, ErasmusMC, Rotterdam, The Netherlands; 5 Department of Molecular Biology and Princeton Neuroscience Institute, Princeton University, Princeton, USA; 6 Departments of Statistics and Neurobiology, University of Chicago, Chicago, USA

## 

The cerebellum is crucial for different types of motor learning. Established theories of cerebellar learning posit that the cerebellum learns by adjusting the weights of Parallel Fiber (PF) to Purkinje cells (PC) synapses, thanks to teaching signals provided by Climbing Fiber inputs. While these theories are consistent with a large body of experimental data, in particular on synaptic plasticity in PF to PC synapses, they cannot easily explain a growing body of experimental work, which seems to indicate a significant role of other sites of plasticity. Recent advances in the development of a large number in transgenic animals, as well as behavioral and electrophysiogical comparative studies between these animals and wild-type animals, have opened an unprecedented window into the mechanisms underlying learning in this structure. In particular, it has been shown that specific knock-outs are impaired selectively on difficult variants of the vestibulo-ocular reflex (VOR) adaptation task, one of the most studied cerebellar-dependent motor learning tasks. These impairments can occur even though the classical plasticity mechanisms are left untouched. These data pose significant new challenges for established models of cerebellar learning.

To better understand the mechanisms of learning in the cerebellum, we built a model that can reproduce the available data on VOR adaptation, in both wild-type and transgenic animals. The model includes some of the main cell types involved in this task: granule cells (GCs), the input layer of cerebellar cortex, that receives vestibular information from the mossy fibers (MFs); Purkinje cells (PCs), as well as molecular layer interneurons (INs); and two cell populations in the medial vestibular nuclei (MVN), one excitatory and one inhibitory, that together control eye movement. The model also includes two sites of learning: the classical GC to PF plasticity site, as well as plasticity in the MF to MVN synapses. We provide a mechanistic understanding on how the system learns VOR adaptation in normal conditions, as well as how the system is impaired by specific knock-outs, which selectively suppress inhibition onto PCs, or increase the excitability of GCs. Finally, we show that our model is consistent with behavioral, as well as in vivo electrophysiological recordings.

